# Type A Aortic Dissection Presenting as Acute Coronary Syndrome in a Young Male Patient: A Case Report

**DOI:** 10.7759/cureus.31578

**Published:** 2022-11-16

**Authors:** Arthur Cecchini, Mohammad H Qureshi, Supriya Peshin, Ahmad Othman, Bhavesh Gajjar

**Affiliations:** 1 Internal Medicine, East Tennessee State University Quillen College of Medicine, Johnson City, USA

**Keywords:** : acute coronary syndrome, acute coronary syndrome, non-st segment elevation myocardial infarction (nstemi), nstemi, type a aortic dissection

## Abstract

Type A aortic dissection (AD) is a devastating cardiovascular emergency requiring emergent surgical intervention. Most patients with AD have several risk factors for the disease including longstanding hypertension, smoking history, atherosclerosis, and old age. Younger patients may also present with AD if a genetic disorder affecting the integrity of the aorta is present. This case presents an otherwise healthy 36-year-old male with no known significant family history who presented with an atypical presentation of aortic dissection. He described a five-day history of chest pressure made worse with exertion followed by progressive dyspnea which prompted him to seek medical attention. His initial laboratory workup revealed an elevated troponin I level which prompted a cardiology consultation in the emergency department. Transthoracic echocardiography revealed dilatation of the aortic root and aortic regurgitation. CT angiography of the chest was performed revealing a type A dissection beginning at the aortic root and terminating proximal to the right brachiocephalic artery. Involvement of the coronary arteries was suspected due to the elevated troponin I. He was taken to the operating room and underwent aortic grafting, right coronary artery bypass, and repair of the left main artery. Unfortunately, at the end of the operation, the patient went into refractory ventricular fibrillation, which progressed to asystole. He was unable to be revived.

## Introduction

Aortic dissection (AD) is a cardiovascular emergency defined by the separation of the layers of the aortic intima and media, allowing the creation of a false lumen in the wall of the aorta. [1,2]. The incidence of AD is estimated to be 2.5 to 15 per 100,000 [1,3]. The Stanford system classifies AD as either type A or type B. Type A dissections involve the ascending aorta and type B dissections include the descending aorta [4]. Risk factors for AD include male gender, older age, hypertension, dyslipidemia, and smoking [1,5,6]. Predisposing conditions include trauma, pregnancy, previous cardiac surgery, stimulant use, and mycotic infection [1]. Loeys-Dietz syndrome, Marfan syndrome, and type-IV Ehlers-Danlos syndrome are also associated with AD, especially in younger patients [1].

AD often presents as acute-onset pain in the chest or back [7]. Painless dissection is less common [8]. Syncope, cerebrovascular accident, heart failure, shock, pericardial effusion, and lower extremity ischemia may also be present [8,9]. Pulse deficits are a poorly sensitive marker or AD. Aortic regurgitation (AR) may occur if dissection into the annulus occurs [9]. D-dimer measurement helps rule out aortic dissection with a negative likelihood ratio of 0.07 [10]. An elevated C-reactive protein (CRP) is a risk factor for in-hospital death and may help predict long-term outcomes [1].

Chest radiography may reveal a widened mediastinum, abnormal aortic contour, or pleural effusion, though its sensitivity is limited [1,9]. CT angiography is the diagnostic study of choice, though transesophageal echocardiography (TEE) may also be performed [1,11]. The sensitivity and specificity for these imaging modalities are high and are estimated to be 98%-100% for CT and 96.8%-100% for TEE [11]. Detection of an intimal flap separating a true and false aortic lumen is diagnostic [1,11].

Anti-impulse therapy, traditionally with a titratable beta-blocker such as esmolol, should be given [2]. Surgical repair is indicated in type A aortic dissection as an extension of the dissection, and complications are likely to occur without emergent management [12].

## Case presentation

A 36-year-old male with no known past medical history presented to the emergency department with chest discomfort which began five days ago. The chest discomfort was described as a non-radiating pressure that was six out of ten in intensity, made worse with exertion, and would resolve after about 15 minutes of rest. He also described shortness of breath over this five-day period, which had progressively gotten worse, prompting him to come to the emergency department. He declined recent illness, strenuous lifting activity, or trauma. The review of systems was otherwise negative.

He declined any family history of connective tissue disorders or cardiovascular disease. He denied tobacco, alcohol, illicit substance, or stimulant use besides light caffeine intake. He did receive his second SARS-CoV-2 booster a week ago.

Vital signs revealed elevated blood pressure and tachycardia (Table 1).

**Table 1:** Vital signs on admission

Vital signs	Patient values
Blood pressure (mmHg)	185/106
Heart rate (per minute)	115
Respiratory rate (per minute)	16
Temperature (degrees Fahrenheit)	98.7
Oxygen saturation (%)	97
Body mass index (kg/m^2^)	34

The physical exam revealed only a grade III diastolic murmur heard best at the left lower sternal boarder (Table 2).

**Table 2:** Physical examination on admission BS: bowel sounds; CN: cranial nerves

Physical examination component	Patient findings
General	Patient sitting in bed comfortably
Head	Atraumatic, normocephalic, eyes and ears midline
Neck	Neck is supple. No jugular venous distention
Cardiac	Tachycardia with a regular rhythm. Grade III Diastolic murmur heard best at the aortic area. No rubs or gallops
Respiratory	Clear to auscultation. No wheezing, crackles, or rhonchi
Abdomen	BS+, soft, nontender to palpation, non distended. No guarding or rebound tenderness
Neurological	No focal deficits noted. CN II - XII grossly intact. Alert and oriented to person, place, time, and situation
Musculoskeletal	No laxity of joints, no joint swelling, and no limited range of motion
Integumentary	Skin was noted to be clean, dry, and intact
Extremities	Peripheral pulses 2+. Upper extremity pulses strong and equal in intensity. Capillary refill less than three seconds and no pedal edema present
Psychological	Normal mood and affect

**Figure 1:**
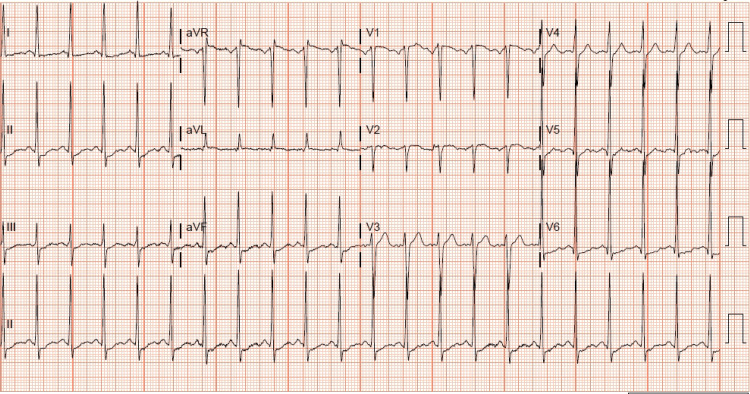
ECG showing sinus tachycardia, upsloping ST wave changes, and left ventricular hypertrophy

An ECG showed sinus tachycardia, upscoping ST wave changes, and left ventricular hypertrophy (LVH) (Figure 1).

The initial laboratory evaluation revealed an elevated troponin I, mild leukocytosis, an elevated CRP, erythrocyte sedimentation rate (ESR), B-type natural peptide (BNP), and D-dimer. His potassium was slightly low (Table 3).

**Table 3:** Laboratory studies on admission

Laboratory studies	Patient values	Reference values
Troponin I (ng/mL)	3.30	0.00-0.02
D-dimer (ng/mL)	753	0-230
C-reactive protein (mg/L)	82.5	0.0-10.0
Erythrocyte sedimentation rate (mm/hr)	41	0-15
B-type natriuretic peptide (pg/mL)	474	0-100
White blood cell count (μL)	10.9	3.5-10.5
Potassium (mEq/L)	3.4	3.5-5.1

**Figure 2:**
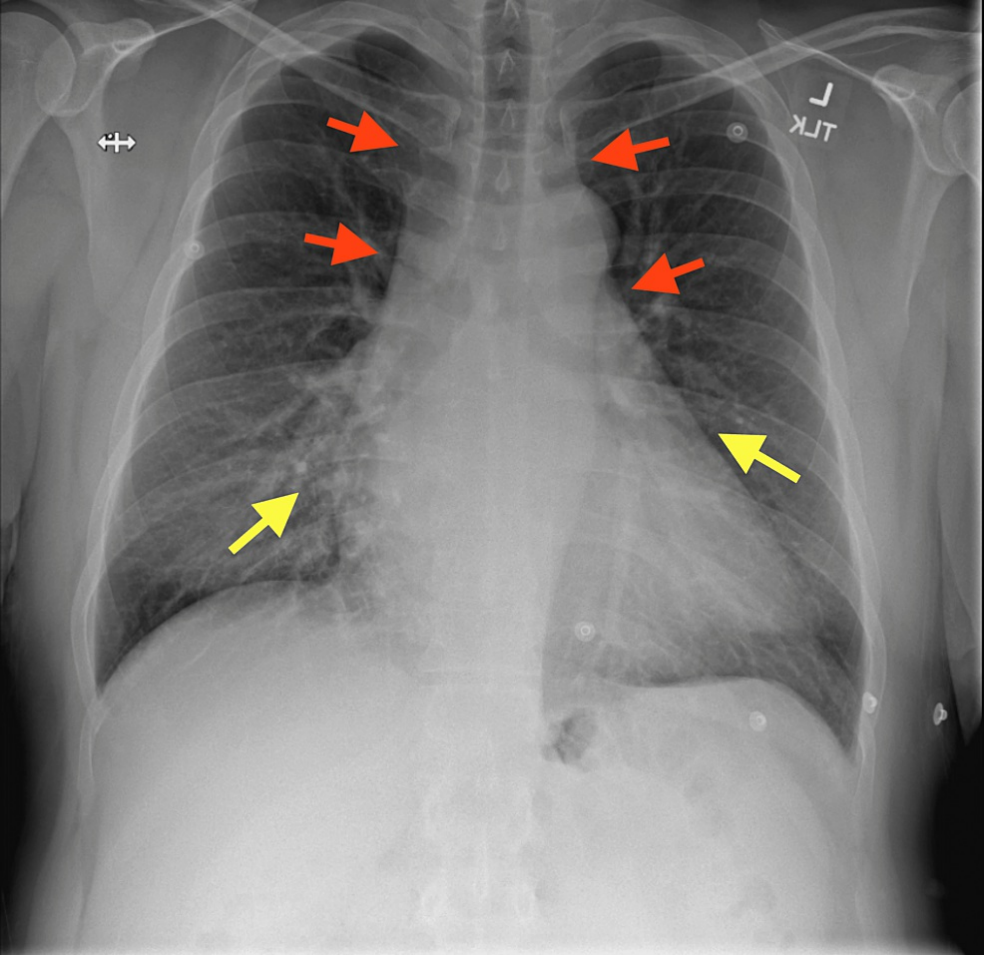
Posterior-anterior chest radiograph showing infrahilar interstitial opacities (yellow arrows), an enlarged cardiomediastinal silhouette (red arrows), and a tortuous thoracic aorta (red arrows)

Posterior-anterior (PA) chest radiograph showed infrahilar interstitial opacities, an enlarged cardio mediastinal silhouette, and a tortuous thoracic aorta (Figure 2).

**Figure 3:**
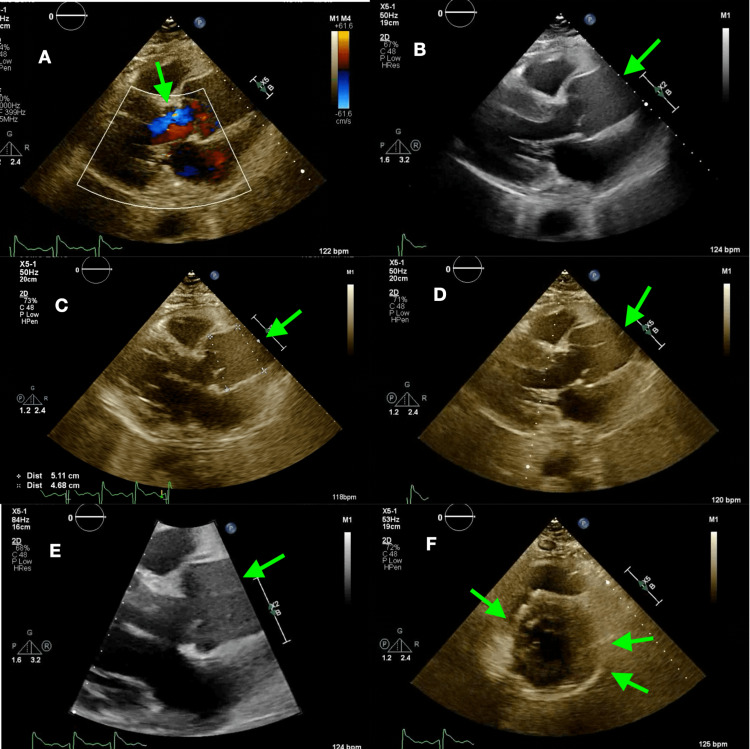
Transthoracic echocardiography showing: A) Severe aortic regurgitation; B) Dilated ascending aorta; C) Measurement of dilated 5.1 cm ascending aorta; D) Dissection flap; E) Close-up view of dilated ascending aorta; F) Increased left ventricular wall thickness

A transthoracic echocardiogram (TTE) was performed showing dilation of the aortic root at 5.1 centimeters, increased left ventricular wall thickness, severe AR, moderate mitral valve regurgitation, mild aortic stenosis, and a left ventricular ejection fraction of 50 to 55%. A dissection flap was noted in the ascending aorta (Figure 3A-3F).

**Figure 4:**
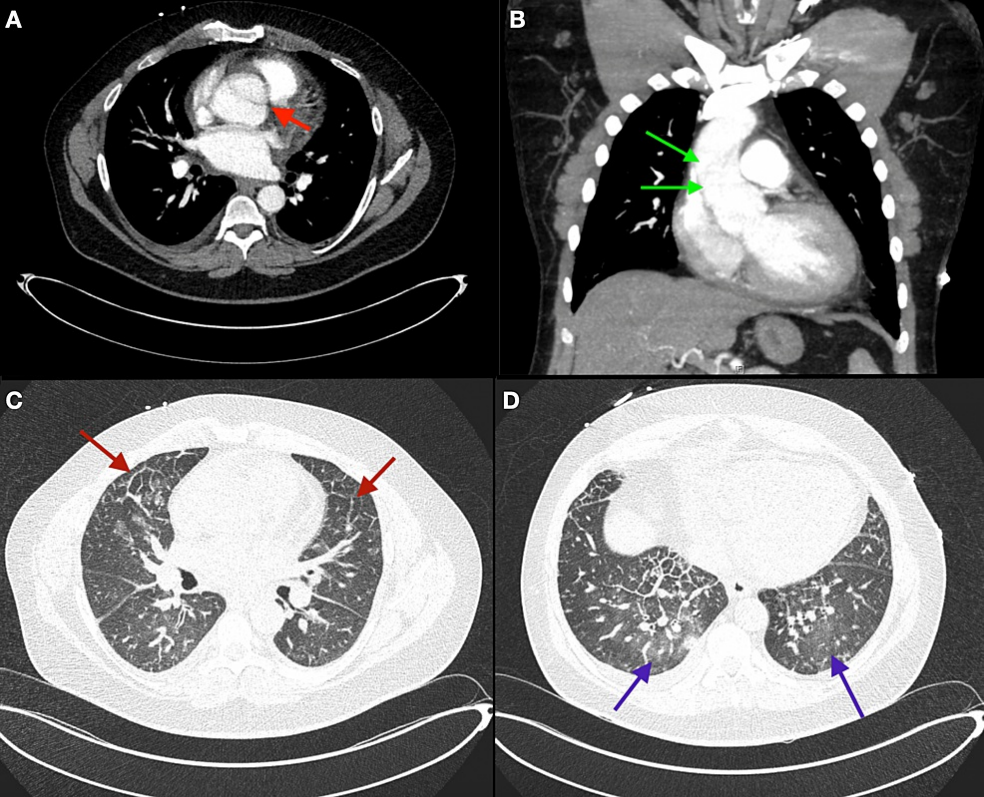
A) Axial view and B) Coronal view arterial phase contrast-enhanced CT of the chest showing a dissection involving the ascending aorta; C) and D) Lung window axial view showing interlobular septal thickening and basilar predominant ground-glass opacities most likely representing pulmonary edema

An arterial phase contrast-enhanced CT of the chest, abdomen, and pelvis was performed showing an aneurysmal-type A thoracic aortic dissection isolated to the ascending aorta, minimal right and left pleural effusion, basilar predominant ground glass opacities, and interlobular septal thickening. The ascending thoracic dissection began at the level of the aortic root and terminated just before the right brachiocephalic artery. The aneurysmal dilation was 3.4 cm at the annulus, 5 x 5 cm at the sinus of Valsalva, 4.7 x 4.7 cm at the sinotubular junction, and 4.8 x 4.8 cm at the level of the pulmonary artery. The rest of the aorta was unremarkable (Figure 4A-4D).

The patient was started on an esmolol drip to reduce his blood pressure and decrease shear stress on the dissecting aorta. He was taken to the operating room emergently and underwent a repair of the type A aortic dissection with a 30 mm gelatin-impregnated aortic root graft. As the right coronary artery was significantly involved, a coronary artery bypass graft was performed, with the saphenous vein being anastomosed to the right posterior descending artery. The left main coronary artery was also involved in the dissection but was able to be repaired via suture placement.

At the end of the operation, the patient went into ventricular fibrillation refractory to repeated defibrillation, amiodarone, lidocaine, and magnesium. This rhythm progressed to asystole, and he was unable to be revived.

## Discussion

According to the International Registry of Aortic Dissection (IRAD), it is estimated that only 7% of aortic dissections occur in patients under 40 years of age [13]. This patient group is more likely to consist of patients with Marfan Syndrome, a bicuspid aortic valve, or previous aortic surgery. They are also less likely to have hypertension [13]. Our patient was atypical as he likely had long-standing hypertension as LVH was noted on both ECG and TTE. He did not have a known history nor clinical examination findings suggestive of a hereditary connective tissue disorder. Our patient also did not use substances associated with AD, have a bicuspid aortic valve, or have a history of aortic surgery.

Our patient also presented in a subacute manner with five days of chest pressure and dyspnea as his primary concerns. This contrasts with much of the current literature describing the most common presentation as abrupt onset chest or back pain of a tearing or stabbing nature, which is estimated to be present in 74% of cases. [8]. The typical description of acute chest pain has a sensitivity of 82.9% and a specificity of 70.7% for AD [1]. We believe that our patient may have dissected into his coronary arteries early in the disease course, leading to his subacute complaint of chest pressure and dyspnea. It is not uncommon for AD to involve the coronary ostium with an estimated 10-15% of patients having ostial involvement [1,14]. His dyspnea was likely also due to his severe AR, which occurs in 40-75% of patients with AD [1]. 

Our patient’s D-dimer was elevated on admission, which is a typical finding seen in AD, as a D-dimer under the reference range carries a negative likelihood ratio of 0.07 [10]. Elevated CRP levels are a risk factor for in-hospital mortality, and our patient’s CRP on admission was eight times the upper limit of normal [1].

It is not uncommon for AD to be mistaken for acute coronary syndrome (ACS), as ACS has an incidence 200 times greater than AD [15], and myocardial ischemia is present in 19.9% of cases [16]. Our patient was initially thought to have ACS, though the diagnosis of AD was suspected when chest radiography revealed a tortuous aorta. The diagnosis was confirmed when an intimal flap was noted on TTE. This prompted a CT angiography of the chest to be performed to determine the extent of involvement. TTE was successful in diagnosing AD in our case, though it only carries an estimated sensitivity of 78-90% [8]. 

Our patient was placed on an esmolol drip for cautious blood pressure reduction while the operating room was prepared for emergent repair. Medical management usually includes the use of an intravenous beta blocker titrated to a goal heart rate of 60 per minute and systolic blood pressure of 100-120 mmHg [2]. Caution in patients such as ours with acute AR should be taken, as an abrupt reduction of heart rate with acute AR may cause severe hypotension [17]. 

As our patient was found to have significant LVH, longstanding hypertension likely led to his AD. He could not recall any family history of cardiovascular disease or predisposing genetic disease. He did not have clinical features of either Marfan syndrome, Loeys-Dietz syndrome, or Ehlers-Danlos syndrome. He had not been to a physician for over 10 years as he was thought to be healthy and suffered a catastrophic AD leading to his demise. 

## Conclusions

This case shows that AD may occur in younger patients without the typical genetic or acquired risk factors for the disease. Patients may present in an atypical manner with progressive symptoms mimicking ACS. Fortunately, suspicion of AD arose when a tortuous aorta was revealed on plain chest radiography films. The case was complicated by severe AR and involvement of multiple coronary arteries which likely lead to his atypical presentation. We surmise that longstanding hypertension was the etiology behind his AD, as evidence of LVH was seen on both ECG and TTE. Overall, this case is a reminder that aortic dissection should remain on the list of differential diagnoses for all patients that present with chest pain.
